# Genotypic Differences in the Effects of Menthol on Nicotine Intake and Preference in Mice

**DOI:** 10.3389/fnins.2022.905330

**Published:** 2022-06-13

**Authors:** Lois S. Akinola, Yumna Rahman, Olivia Ondo, Jada Gonzales, Deniz Bagdas, Asti Jackson, Nicole Davidson-Wert, M. Imad Damaj

**Affiliations:** ^1^Department of Pharmacology and Toxicology, Medical College of Virginia, Virginia Commonwealth University, Richmond, VA, United States; ^2^Department of Psychiatry, School of Medicine, Yale University, New Haven, CT, United States; ^3^Yale Tobacco Center of Regulatory Science, Yale School of Medicine, New Haven, CT, United States

**Keywords:** menthol, nicotine, two-bottle choice, DBA, C57BL/6, mice, conditioned place preference, genetics

## Abstract

Menthol has been shown to exacerbate elements of nicotine addiction in humans and rodents; however, the mechanisms mediating its effects are not fully understood. This study examined the impact of genetic factors in menthol’s effects on oral nicotine consumption by comparing two inbred mouse strains with differing sensitivities to nicotine. C57BL/6J (B6J) mice are nicotine-preferring, while DBA/2J (D2J) mice are not. While the effects of menthol on oral nicotine consumption have been highlighted in B6J mice, it is unknown if they extend to the D2J strain as well. Consequently, adolescent (PND 21) and adult (PND 63), male and female D2J mice were subjected to the nicotine two-bottle choice (2BC) paradigm with orally and systemically administered menthol. Then, we evaluated its impact on nicotine pharmacological responses in conditioned reward and nociception after systemic administration and, lastly, investigated the potential involvement of the TAAR1 gene and α7 nAChRs in menthol’s effects. Menthol failed to enhance oral nicotine consumption in adult and adolescent female and male D2J mice. Moreover, this lack in effect was not due to nicotine concentration, oral aversion to menthol, or basal preference for nicotine. Menthol also failed to augment nicotine reward or enhance nicotine-induced antinociception in D2J mice, demonstrating that genetic background plays a significant role in sensitivity to menthol’s effects on nicotine. Furthermore, TAAR1 or α7 nAChRs did not seem to mediate menthol’s differential effects in D2J mice. These findings support the existence of genotype-specific mechanisms that may contribute to the variable effects of menthol in different populations.

## Introduction

Although the harmful effects of persistent nicotine use have been well-documented, many users still face immense difficulty remaining abstinent and quitting permanently due to its addictive properties (Hyland et al., 2006; Yong et al., 2014; Chaiton et al., 2018). Moreover, accumulating evidence suggests that in addition to increasing the appeal and willingness to use tobacco products in general, certain flavorants may propagate nicotine dependence by exerting potent pharmacological effects (Ferris Wayne and Connolly, 2004; Henderson et al., 2017; Avelar et al., 2019; Cooper et al., 2020, 2021; Palmatier et al., 2020; Patten and De Biasi, 2020). Among such is menthol, a commonly used flavoring in tobacco products and the sole exclusion to the flavor ban in cigarettes under the U.S. Family Smoking and Prevention Act and recently in the restriction of flavored cartridge-based electronic nicotine delivery systems (U.S. Food and Drug Administration, 2011). Although cigarette smoking is the most common form of tobacco use, menthol is also used in other tobacco products like cigars, and smokeless tobacco products like chewing tobacco, snuff, snus, dissolvable tobacco products, and electronic cigarettes (Alpert et al., 2008; Levy et al., 2011; Villanti et al., 2017; Odani et al., 2020).

Menthol use in tobacco products has been shown to exacerbate elements of nicotine addiction. In general, smokers of mentholated cigarettes report higher dependence scores, higher relapse rates, and poorer cessation outcomes compared to non-menthol smokers (Wickham, 2015; Wickham, 2020). Evidence shows that menthol masks the harsh orosensory effects of nicotine and produces respiratory suppressive effects to the irritants contained in cigarette smoke, thus facilitating the palatability of cigarettes (Garten and Falkner, 2004; Willis et al., 2011). The results have also been paralleled in rodent studies where menthol has been shown to decrease oral aversion to nicotine (Fan et al., 2016), enhance intravenous nicotine self-administration (Palmatier et al., 2020), and nicotine reward and withdrawal in rodents (Alsharari et al., 2015; Henderson et al., 2017). In recent studies, we demonstrated that orally and systemically administered menthol enhances oral nicotine consumption in B6J mice in a sex-, age-, and concentration-dependent manner using the 2BC test, suggesting the involvement of both orosensory-dependent and independent mechanisms in menthol’s effects (Bagdas et al., 2020). Some evidence highlights distinct biological mechanisms for how menthol can alter nicotine consumption, including modulation of nicotinic receptor function and expression through allosteric mechanisms (Hans et al., 2012; Ashoor et al., 2013; Brody et al., 2013), alterations in dopamine signaling in the central nervous system (Henderson et al., 2017), and changes in nicotine metabolism (Benowitz et al., 2004; Alsharari et al., 2015; Ha et al., 2015). In addition, genetic factors may also play a role in sensitivity to menthol’s effects on nicotine.

Population studies indicate inter-individual and inter-ethnic differences in preference for mentholated cigarettes (Giovino et al., 2004; Wickham, 2015). For example, young individuals, African Americans, females, and lower socioeconomic status smokers are more likely to smoke mentholated cigarettes. While environmental and socioeconomic factors have been offered as a possible explanation for observed differences in menthol preference, including the influence of targeted advertising and promotions (Cummings et al., 1987; Landrine et al., 2005), genetic contributions to individual differences in menthol preference are also plausible. Importantly, studies indicate that a large percentage of the variance associated with smoking initiation and maintenance results from genetic factors (Vink and Boomsma, 2011). For example, an allelic variant in the transient receptor potential Ankyrin 1 (TRPA1) receptor, a menthol target, is associated with an increased preference for mentholated cigarettes in heavy smokers (Uhl et al., 2011). Another study uncovered a variant in the MRGPRX4 gene, encoding a G-protein coupled receptor expressed in sensory neurons (Kozlitina et al., 2019), showing that this haplotype contributes to differences in preference for mentholated cigarettes, supporting the existence of genetic factors predisposing vulnerable populations to menthol cigarette smoking.

Comparative studies of phenotypes expressed by different inbred strains of mice could potentially identify biological and genetic determinants of variability in menthol sensitivity at preclinical levels. Inbred mouse lines are routinely used to study the genetics of particular phenotypes of interest (Baud and Flint, 2017; Kirkpatrick et al., 2017; Bryant et al., 2020). Of particular interest to our studies is the BXD line, a family of recombinant inbred strains derived by crossing B6J and D2J and inbreeding their progeny for 20 or more generations. To our advantage, B6J and D2J mouse strains differ significantly in their sensitivity to the effects of nicotine (Siu and Tyndale, 2007; Kutlu et al., 2015; Bagdas et al., 2019). B6J mice appear to be a nicotine-preferring inbred strain of mice, while D2J mice are non-nicotine-preferring. However, it is unknown if these differences extend to their sensitivity to menthol-induced enhancement of nicotine intake. We, therefore, aimed to compare the impact of menthol on nicotine intake in these two strains. Indeed, differences in menthol sensitivity between these two strains might indicate genetically determined differences in this phenotype and could justify further study in the BXD lines that would allow investigation of genetic mapping of these traits.

While we have previously highlighted the effects of menthol on nicotine consumption in B6J mice (Bagdas et al., 2020), this study aimed to assess menthol’s effects in D2J mice. In the current study, we first investigated the impact of menthol (in solution) on oral nicotine consumption in adult and adolescent male and female D2J mice in the 2BC paradigm. Then, menthol was administered systemically to parse out the extent to which orosensory and non-orosensory factors contributed to its effects in oral nicotine consumption. We also examined if the effects of menthol were dependent on basal nicotine preference. Select lines of BXD mice with differing preferences for oral nicotine (10–90%) were subjected to the nicotine 2BC paradigm with menthol. Then, we evaluated the impact of menthol on other nicotine pharmacological responses in conditioned reward and nociception after systemic administration in the conditioned place preference (CPP) and hot plate tests, respectively. Furthermore, to investigate the potential mechanisms underlying menthol’s effects in D2J mice, we evaluated the role of the trace amine-associated receptor 1 (TAAR1) gene in menthol’s effects in D2J mice by comparing oral nicotine consumption in D2J mice to DBA/2NCrl (D2N) mice, a substrain from Charles River Laboratories that lacks the mutation in the TAAR1 gene that D2J mice possess. Indeed, this spontaneous mutation in the TAAR1 gene has been found to impact methamphetamine drinking exclusively in D2J mice but not in other DBA/2 substrains (Reed et al., 2018). Lastly, we investigated the potential role of the α7 nAChR subunit in menthol’s effects in D2J mice. α7 nAChRs play a key role in nicotine-related reinforcement and reward (Nomikos et al., 2000; Brunzell and McIntosh, 2012); and we have also previously shown that they play a modulatory role in the effects of menthol on oral nicotine consumption in B6J mice (Bagdas et al., 2020). Since D2J mice have a higher basal mRNA expression of α7 nAChR subunit in the nucleus accumbens (NAc) compared to B6J mice, we thus examined if this increased basal tone contributed to their nicotine-avoidant phenotype using the selective α7 nAChR antagonist, methyllycaconitine (MLA), as a tool.

## Materials and Methods

### Animals

Male and female adult (PND 63-70 at the beginning of the study) and adolescent (PND 21 at the beginning of the study) mice were used. DBA/2J (D2J), C57BL/6J (B6J), and BXD (BXD53/2RwwJ, BXD55/RwwJ, BXD62/RwwJ, BXD64/RwwJ, BXD65/RwwJ, and BXD65a/RwwJ) mice were purchased from Jackson Laboratory (Bar Harbor, ME, United States), while DBA/2NCrl (D2N) mice were purchased from Charles River Laboratories (Wilmington, MA, United States). D2J, D2N, and BXD mice were used in drinking studies, while B6J mice were used only in CPP studies. For drinking studies, upon arrival, adult and adolescent mice were individually housed in a temperature- and humidity-controlled out-of-vivarium space (21 ± 3°C, 55 ± 10%) on a 12-h light/dark cycle for the duration of the study. For all drinking studies, mice were given *ad libitum* food but constantly received their water as drinking solutions from two bottles. This study was approved by the Institutional Animal Care and Use Committee of Virginia Commonwealth University. All studies were carried out in accordance with the National Institutes of Health Guidelines for the Care and Use of Laboratory Animals.

### Drugs and Chemicals

(-)-Nicotine base liquid [(-)-1-Methyl-2-(3-pyridyl) pyrrolidine, (S)-3-(1- Methyl-2-pyrrolidinyl) pyridine)] (cat no: N3876), (-)-nicotine hydrogen tartrate salt [(-)-1-methyl-2-(3- pyridyl) pyrrolidine (þ)-bitartrate salt] (cat no: SML1236), methyllycaconitine citrate salt (MLA) [1α,4(S),6β,14α,16β]-20-Ethyl-1,6,14,16-tetramethoxy-4-[[[2-(3-methyl-2,5-dioxo-1-pyrrolidinyl)benzoyl]oxy]methyl]aconitane-7,8-diol citrate salt] (cat no: M168) and (-)-menthol [5-Methyl-2-(1-methylethyl)cyclohexanol] (cat no: W266523) were purchased from Sigma-Aldrich (St. Louis, MO, United States). For drinking studies, all solutions (nicotine, menthol, and mentholated nicotine) were prepared in deionized water (DI) without any sweeteners and replaced every three days. While nicotine base liquid was used for drinking studies, nicotine hydrogen tartrate salt was used for systemic injections. In conditioned place preference studies, menthol was administered intraperitoneally (i.p.) while nicotine was given subcutaneously (s.c.). For i.p. injections, menthol was dissolved in a mixture of 1:1:18 [1 vol ethanol/1 vol Kolliphor EL (Sigma-Aldrich, St. Louis, MO, United States)/18 vol distilled water], while nicotine tartrate salt and MLA were dissolved in physiological saline for s.c. injections. All injections were administered at a total volume of 1mL/100 g body weight, and nicotine doses are expressed as the free base of the drug.

### Two-Bottle Choice Test

The two-bottle choice (2BC)test is a technically simple, robust, and high throughput behavioral assay that has been used extensively to study the biological and behavioral effects of drugs of abuse in rodents, including nicotine (Meliska et al., 1995; Aschhoff et al., 2000; Todte et al., 2001; Nesil et al., 2011). We used this paradigm as previously described (Bryant et al., 2019; Bagdas et al., 2020). Briefly, animals were individually housed and given *ad libitum* access to DI water for a week from two 25 mL serological pipettes modified into sipper tubes to habituate them to the new drinking conditions. Following habituation, animals were then presented with a choice of DI water and a test solution (solution varied depending on the experiment). Fluid consumption was then monitored over a 24-h period for several days to assess the animal’s relative preference for the test solution over water. Our studies assessed solutions containing either nicotine alone, menthol alone, or a combination of nicotine and menthol. In addition, mice were provided with unsweetened solutions to avoid confounds such as the potential reinforcing effects of sweeteners. Primary outcomes were drug intake and drug preference (depending on the experiment). Intake was calculated as mg of drug per kilogram (mg/kg) of body weight and preference as the volume of drug solution consumed as a percentage of the total fluid consumed. Fluid intake was recorded within the light cycle and at the same time daily, and bottle positions were alternated each day to prevent the development of a side preference. Bodyweight measurements were taken at the beginning of each experiment and every other day; the averaged mass was then used to calculate daily nicotine intake for each concentration. An empty animal-free cage with two bottles, including the test solution and DI water, was used to determine the spillage volume from gravity alone. The average spillage volume was ∼0.2 mL per day.

For drinking studies, five types of experiments were performed:

*Concentration-response of oral menthol consumption in DBA/2J mice*. Male and female adult and adolescent D2J mice were given a choice to self-administer either water or menthol solution in the 2BC paradigm in a within-subject manner. Menthol concentrations ranged from 30 to 210 μg/mL, with increments in concentration every three days. This constraint was enforced to ensure that adolescent animals did not surpass the early-to-mid-adolescent period so that animals were within the range of sexual maturation but without behavioral and psychopharmacological development. Consequently, adolescents were only tested during their second week of peri-adolescence (PND 22-34) through early mid-adolescence (PND 35-47) (Brust et al., 2015).

*Effect of menthol on oral nicotine consumption*. The effects of menthol (administered in drinking solution) on oral nicotine consumption were assessed in three different strains of mice. In one cohort, male and female adult and adolescent D2J mice were given a choice of water or mentholated nicotine with low (10 μg/mL), moderate (60 μg/mL), or high (200 μg/mL) nicotine concentrations. In each group, nicotine was kept constant while menthol concentrations were varied (0–120 μg/mL). We included a range of nicotine to capture the effects of menthol at concentrations likely to be tolerated and concentrations likely to be aversive. The nicotine concentrations chosen are based on our previous studies in B6J mice showing modest preference at 10 and 60 μg/mL and aversion around 200 μg/mL (Bagdas et al., 2019). In separate cohorts, adult D2N and BXD mice were also subjected to the same paradigm with limited dosing (Supplementary Material). Adult male and female D2N mice were given a choice of DI water or menthol + nicotine. Nicotine was kept constant at 10 μg/mL while menthol concentration was varied (0, 10 & 60 μg/mL). For BXD animals, six lines (BXD53/2RwwJ, BXD55/RwwJ, BXD62/RwwJ, BXD64/RwwJ, BXD65/RwwJ, and BXD65a/RwwJ) of female animals were used. BXD mice were given a choice of DI water or menthol + nicotine. Nicotine was kept constant at 60 μg/mL while menthol concentration was varied (0, 30, and 90 μg/mL) and increased every three days.

*Effect of systemically administered menthol on oral nicotine consumption in DBA/2J mice*. To determine the extent to which orosensory factors alone contribute to the impact of menthol on nicotine consumption in D2J mice, animals were administered menthol intraperitoneally while subjected to nicotine 2BC. Adult male and female mice were given a choice of water or nicotine solutions (60 or 200 μg/mL) for seven days. On days 1–5, mice were poked daily to simulate i.p. injections, then on day 6 (pre), mice received an acute injection of menthol (0.1, 1, or 10 mg/kg). Nicotine intake and preference were then measured 24 hr. after injection on day 7 (post).

*Nicotine concentration-response in DBA/2NCrl mice.* Adult male and female D2N mice were assessed in a nicotine concentration-response in the 2BC test. Mice were given a choice of water or nicotine (10–240 μg/mL) in a within-subject manner. Nicotine concentrations were increased every 3 days (Supplementary Material).

*Effect of acute MLA on oral nicotine consumption in DBA/2J mice.* Adult male and female D2J mice were given a choice of water or nicotine (10 μg/mL) for 5 days. On day 5, mice received a subcutaneous injection of either saline or MLA (10 mg/kg) in the AM and PM, 4 h apart. Nicotine consumption was then measured after 24 h (Supplementary Material).

### Conditioned Place Preference Test

As previously described, an unbiased CPP paradigm was performed (Kota et al., 2007). The CPP apparatus (Med Associates, St. Albans, VT, United States, ENV3013) consisted of three linear chambers: a white, black, and neutral chamber, which differed in overall color and floor texture (wire mesh, grid rod, and smooth PVC), respectively. The white and black chambers (16.76 cm × 12.7 cm × 12.7 cm) are separated by a narrow gray (neutral) chamber (9.78 cm × 12.7 cm × 12.7 cm). Partitions could be removed to allow access from the gray chamber to the black and white chambers. Both adult D2J and B6J mice were used in these studies. On day 1, animals were confined to the middle gray chamber for a 5 min habituation and then allowed to move freely between all three chambers for 15 min. Time spent in each chamber was recorded, and these data were used to populate groups of approximately equal bias in baseline chamber preference. Twenty-minute conditioning sessions occurred twice a day (days 2–4). During conditioning sessions, mice were confined to one of the larger (black or white) chambers. The vehicle group received saline in one large chamber in the morning and saline in the other chamber in the afternoon. The nicotine group received nicotine (0.1 mg/kg, s.c.) in one large chamber and saline in the other chamber. Treatments were counterbalanced equally to ensure that some mice received the unconditioned stimulus in the morning while others received it in the afternoon. The nicotine-paired chamber was randomized among all groups. Sessions were four hours apart and were conducted by the same investigator with an unblinded design. On each of the conditioning days, mice were pretreated with menthol (0.1 or 0.3 mg/kg, i.p.) or its vehicle (1:1:18) 30 min before nicotine (0.1 mg/kg, s.c.), or saline injection. On the test day (day 5), mice were allowed access to all chambers for 20 min in a drug-free state. The preference score was calculated by determining the difference between the time spent in the drug-paired side during test day versus the baseline day. A positive score indicates a preference for the drug-paired side, whereas a negative number indicates an aversion to the drug-paired side. A number at or near zero indicates no preference for either side.

### Hot Plate Test

As a measure of antinociception, the hot plate test was evaluated. Mice were placed onto a hot plate (Thermojust Apparatus) maintained at 55°C and surrounded by a 10 cm wide glass cylinder as described by Eddy and Leimbach (1953) and Atwell and Jacobson (1978). Two baseline latencies at least ten min apart were determined for each mouse. The baseline latency (reaction time) ranged from eight to twelve seconds and was the time spent on the hot plate before showing signs of nociception (e.g., jumping, paw licks). To avoid tissue damage, a cut-off latency of 20 s was imposed. Adult D2J mice were injected intraperitoneally with menthol (100 mg/kg) or vehicle (1:1:18), then received nicotine (0, or 1.5 mg/kg, s.c.) 30 min later and were tested 5-, 30-, 60- and 120-min post-injection. Antinociceptive response was calculated as percent maximum possible effect (% MPE), where % MPE = [(test-baseline)/(20-baseline) × 100].

### Statistical Analysis

All data were analyzed using the GraphPad software, version 9.3.1 (GraphPad Software, Inc., La Jolla, CA, United States) and expressed as the mean ± SEM. Normality and equal variance were verified by the Shapiro–Wilk and Brown–Forsythe tests, respectively. Drinking studies were performed using a within-subject design. Statistical analyses were then conducted using an ordinary or repeated measures (RM) two- or three-way analysis of variance (ANOVA) test followed by a *post hoc* test where appropriate. For ANOVA tests using repeated measures, a Geisser-Greenhouse correction was used where appropriate (alpha 0.05) to correct for data with unequal variances, resulting in F values being reported with decimals. A three-way ANOVA with repeated measures was used to determine the overall interaction of the three factors (age, sex, and menthol concentration) in all 2BC tests. An ordinary three-way ANOVA was used to determine the overall interaction of the three factors (strain, nicotine treatment, and menthol treatment) in the CPP test. In addition, a RM two-way ANOVA with time and treatment as factors was used to determine the difference between treatment groups at two time-points (Pre and Post) in the nicotine 2BC study with i.p. menthol administration and in the hot plate test. For Supplementary Data, a RM two-way ANOVA test (drinking studies and hot plate test) followed by a Dunnett’s or Sidak *post hoc* test where appropriate, except data on the BXD mice, which was analyzed using the non-parametric Friedman test. All differences were considered significant when *P* < 0.05.

## Results

### Adult and Adolescent DBA/2J Mice Consume Menthol in an Age-, Sex- and Concentration-Dependent Manner

**FIGURE 1:**
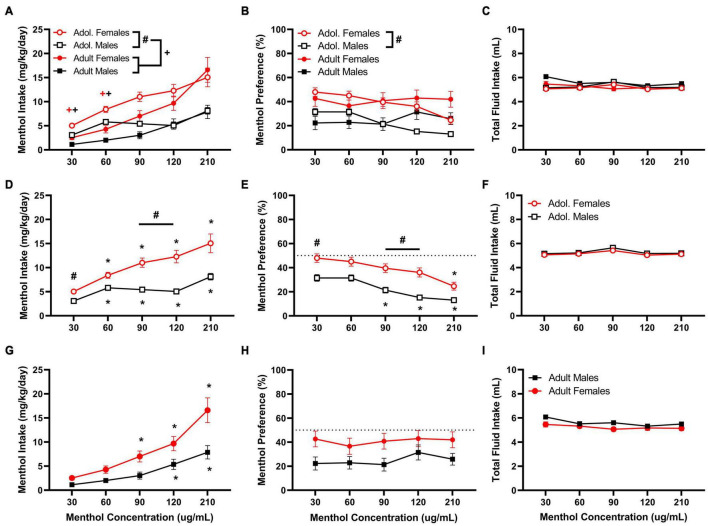
Oral menthol intake and preference in adolescent and adult male and female DBA/2J mice. Mice were given a choice of DI water or menthol solutions (30–210 μg/mL) in a within-subject manner in the two-bottle choice paradigm. Concentrations of menthol increased every three days, and the averaged values from all 3 days were used to calculate menthol intake and preference. Intake is calculated as mg of drug per kilogram (mg/kg) of body weight and preference as the volume of drug solution consumed as a percentage of the total fluid consumed. **(A,D,G)** Average menthol intake (mg/kg/day), **(B,E,H)** menthol preference (%), and **(C,F,I)** total fluid intake are shown. Data are presented as the mean ± SEM of 10–20 mice/sex/age. Results are based on a RM three-way ANOVA with age, sex, and menthol concentration as factors. ^+^*P* < 0.05 vs. adults. *P < 0.05 vs. 30 μg/mL menthol solution. ^#^*P* < 0.05 vs. males.

First, we established a concentration-response of oral menthol consumption in adolescent and adult male and female D2J mice. The results are presented in Figure 1. Data analysis reveals significant age, sex, and concentration effects on menthol intake. In general, females and adolescent mice consume more menthol in solution indicated by their markedly higher intake compared to their male counterparts. A RM three way-ANOVA with age, sex and menthol concentration as factors revealed significant effects of sex and menthol concentration for both intake [F_*intake:sex*_ (1, 161) = 31.79, *P* < 0.0001; F_*intake:concentration*_ (2.109, 339.5) = 60.76, *P* < 0.0001; Figure 1A] and preference outcomes [F_*preference:sex*_ (1, 161) = 20.44, *P* < 0.0001; F_*preference:concentration*_ (3.746, 603.1) = 7.209, *P* < 0.0001; Figure 1B], but revealed significant effects of age only in menthol intake [F_*intake:age*_ (1, 161) = 6.130, *P* = 0.0143; Figure 1A]. It also revealed significant interactions between concentration and age factors [F_*intake:concentrationxage*_ (4,644) = 4.403, *P* = 0.0016] and concentration and sex factors [F_*intake:concentrationxsex*_ (4,644) = 8.378, *P* < 0.0001] for menthol intake, but showed only a significant interaction between concentration and age factors [F_*preference:concentrationxage*_ (4,644) = 15.39, *P* < 0.0001] for menthol preference. The three-way ANOVA, however, revealed no significant interaction among all three factors for either menthol intake or preference outcomes, and no differences in total fluid intake were observed in any of the groups (Figure 1C).

A Tukey *post hoc* test revealed significant age differences in menthol intake when comparing adult and adolescent animals at low (30 and 60 μg/mL) but not at high concentrations (90,120, or 210 μg/mL), such that adolescent mice show a higher intake for menthol than adult animals (Figure 1A). For sex-related effects, significant differences were observed only in adolescent mice at 30, 90, and 120 μg/mL in both menthol intake and preference, but not in adult animals (Figures 1D,E), demonstrating that female adolescent mice consume more menthol than their male and adult counterparts. Furthermore, in adolescent mice, the *post hoc* test revealed significant increases in menthol intake at all concentrations compared to the lowest concentration tested (30 μg/mL) in both sexes (Figure 1D), but only at 90, 120, and 210 μg/mL in males and at 120 and 210 μg/mL in females for menthol preference (Figure 1E). No differences were observed in total fluid intake between adolescent male and female animals (Figure 1F).

In adult animals, the Tukey *post hoc* test revealed no significant effects of sex on menthol intake or preference but reported significant effects of menthol concentration on menthol intake at 120 and 210 μg/mL for males and at 90, 120, and 210 μg/mL for female animals (Figure 1G). However, no effects of menthol concentration on menthol preference were observed in adult male or female animals (Figure 1H). Also, no differences were observed in total fluid intake between male and female animals (Figure 1I).

### Menthol Does Not Enhance Nicotine Consumption in Adult or Adolescent DBA/2J Mice

**FIGURE 2:**
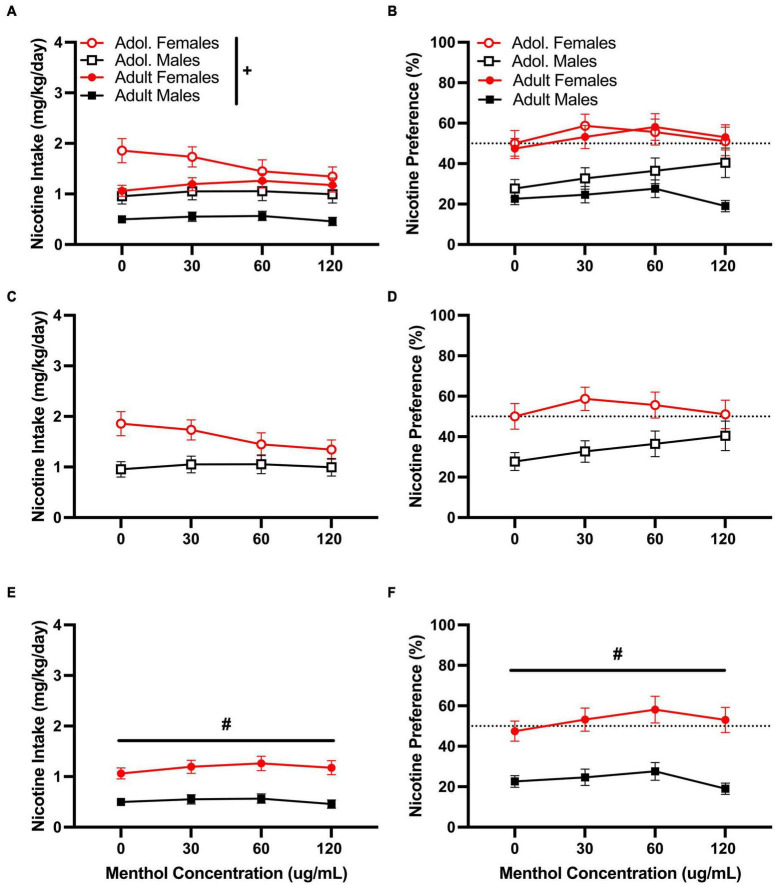
Effects of menthol on oral nicotine (10 μg/mL) consumption in adult and adolescent male and female DBA/2J mice. Mice were given a choice of DI water or menthol + nicotine [nicotine (10 μg/mL) + menthol 0–120 μg/mL] in a within-subject manner in the two-bottle choice paradigm. Intake is calculated as mg of drug per kilogram (mg/kg) of body weight and preference as the volume of drug solution consumed as a percentage of the total fluid consumed. **(A,C,E)** Average nicotine intake (mg/kg/day) and **(B,D,F)** nicotine preference (%) are shown. Data are presented as the mean ± SEM of 10–15 mice/sex/age. Results are based on a RM three-way ANOVA with age, sex, and menthol concentration as factors. ^+^*P* < 0.05 vs. adults. *P < 0.05 vs. 0 μg/mL. ^#^*P* < 0.05 vs. males.

Menthol failed to enhance oral nicotine consumption in adult or adolescent D2J mice at any nicotine concentrations tested. However, it decreased rather than increased nicotine consumption. When assessed, a RM three way-ANOVA of the low nicotine concentration (10 μg/mL) with age, sex, and menthol concentration, revealed significant effects of sex in both nicotine intake [F_10μ*g/mL:intake:sex*_ (1,116) = 34.05, *P* < 0.0001] and preference [F_10μ*g/mL:preference:sex*_ (1,116) = 32.82, *P* < 0.0001] but only a significant effect of age [F_10μg/mL:intake:age_ (1, 116) = 18.67, *P* < 0.0001] in nicotine intake (Figure 2). No overall effects of menthol concentration were reported for either nicotine intake or nicotine preference. When compared, a Tukey *post hoc* test revealed no significant effects of age at any of the menthol concentrations tested for either male or female animals. However, it did report significant sex differences only in adult animals at all menthol concentrations tested.

**FIGURE 3:**
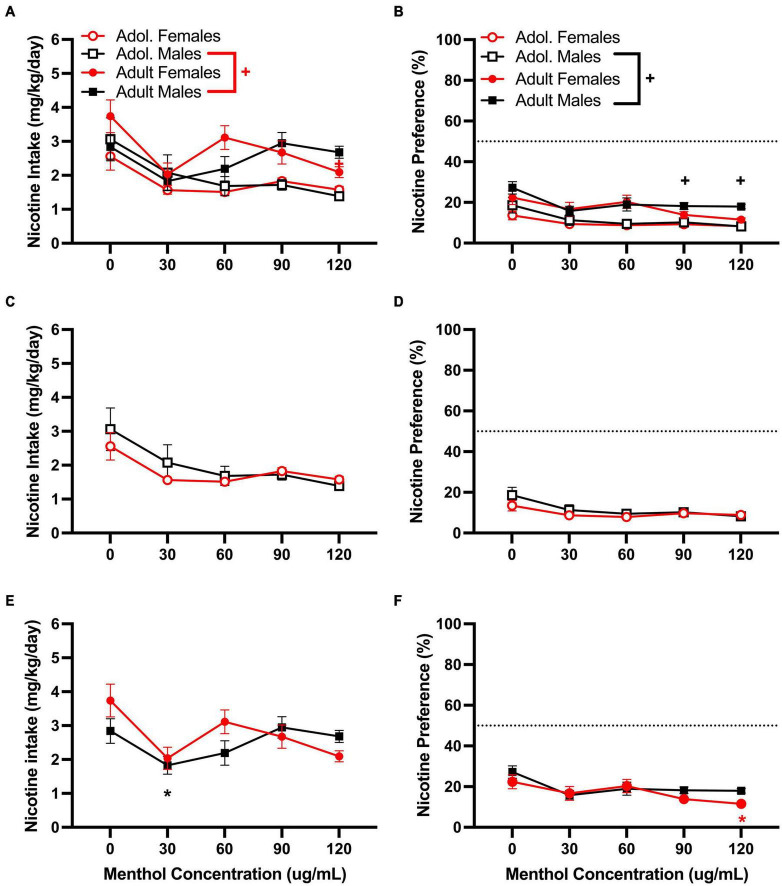
Effects of menthol on oral nicotine (60 μg/mL) consumption in adult and adolescent male and female DBA/2J mice. Mice were given a choice of DI water or menthol + nicotine [nicotine (60 μg/mL) + menthol 0–120 μg/mL] in a within-subject manner in the two-bottle choice paradigm. Intake is calculated as mg of drug per kilogram (mg/kg) of body weight and preference as the volume of drug solution consumed as a percentage of the total fluid consumed. **(A,C,E)** Average nicotine intake (mg/kg/day) and **(B,D,F)** nicotine preference (%) are shown. Data are presented as the mean ± SEM of 10-15 mice/sex/age. Results are based on a RM three-way ANOVA with age, sex, and menthol concentration as factors. ^+^*P* < 0.05 vs. adults. *P < 0.05 vs. 0 μg/mL. ^#^*P* < 0.05 vs. males.

For the moderate nicotine concentration (60 μg/mL), a RM three way-ANOVA with age, sex and menthol concentration, revealed significant effects of age and menthol concentration on nicotine intake [F_60μ*g/mL:intake:age*_ (1, 146) = 8.775, *P* = 0.0036; F_60μ*g/mL:intake:concentration*_ (2.902, 365.6) = 10.97, *P* < 0.0001; Figure 3A] and nicotine preference [F_60μ*g/mL:preference:age*_ (1, 161) = 17.92, *P* < 0.0001; F_60μ*g/mL:preference:concentration*_ (2.832, 399.3) = 12.12, *P* < 0.0001; Figure 3B] but not sex, for the moderate nicotine (60 μg/mL) concentration (Figure 3). No significant interactions of all three factors (age, sex, or menthol concentration) were reported by the ANOVA for either nicotine intake or nicotine preference; however, a significant interaction of the concentration and sex factors was observed only for nicotine intake [F_60μ*g/mL:intake:concentrationxsex*_ (4, 504) = 2.453, *P* < 0.0451, Figure 3A]. For age-related effects, a Tukey *post hoc* test revealed no significant differences in nicotine intake between adult or adolescent male mice at any of the menthol concentrations tested but showed significant age differences between female adult and adolescent animals only at 120 μg/mL (Figure 3A). The results were converse for the nicotine preference outcome. The *post hoc* test showed significant age-related effects in male animals only at 90 and 120 μg/mL, but not in female animals at any of the menthol concentrations tested (Figure 3B). For menthol concentration-related effects in nicotine intake, the Tukey *post hoc* analysis showed significant effects of menthol concentration only at 30 μg/mL in adult male animals compared to 0 μg/mL. It revealed no significant effects of menthol concentration in adolescent male or female animals or adult females (Figures 3C,E); only an effect at 120 μg/mL was observed in adult female animals for nicotine preference. No significant effects of menthol concentration on nicotine preference were observed in either adolescent male or female animals or adult male animals (Figures 3D,F).

**FIGURE 4:**
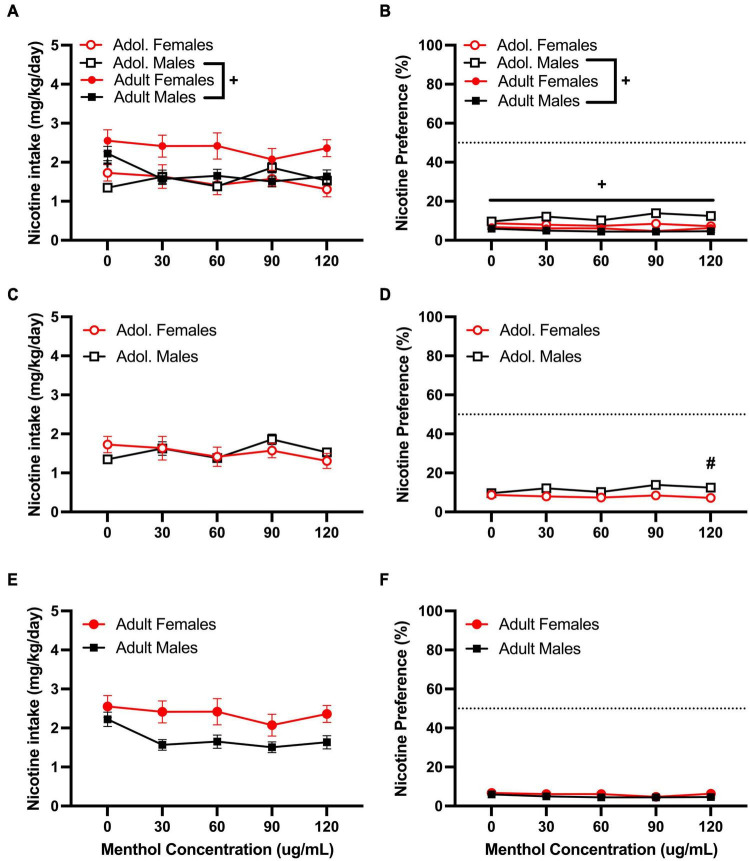
Effects of menthol on oral nicotine (200 μg/mL) aversion in adult and adolescent male and female DBA/2J mice. Mice were given a choice of DI water or menthol + nicotine [nicotine (200 μg/mL) + menthol 0–120 μg/mL] in a within-subject manner in the two-bottle choice paradigm. Intake is calculated as mg of drug per kilogram (mg/kg) of body weight and preference as the volume of drug solution consumed as a percentage of the total fluid consumed. **(A,C,E)** Average nicotine intake (mg/kg/day) and **(B,D,F)** nicotine preference (%) are shown. Data are presented as the mean ± SEM of 10–15 mice/sex/age. Results are based on a RM three-way ANOVA with age, sex, and menthol concentration as factors. ^+^*P* < 0.05 vs. adults. *P < 0.05 vs. 0 μg/mL. ^#^*P* < 0.05 vs. males.

We then tested the ability of menthol to decrease oral aversion to nicotine by testing a higher nicotine (200 μg/mL) concentration (Figure 4). We have previously shown a strong aversion to nicotine at this concentration in both D2J and B6J mice (Bagdas et al., 2019). When assessed, a RM three way-ANOVA with age, sex and menthol concentration as factors revealed significant effects of age [F_200μ*g/mL:intake:age*_ (1, 86) = 10.45, *P* = 0.0015; F_200μ*g/mL:preference:age*_ (1, 86) = 75.81, *P* < 0.0001] and sex [F_200μ*g/mL:intake:sex*_ (1, 86) = 4.141, *P* = 0.0449; F_200μ*g/mL:preference:sex*_ (1, 86) = 6.800, *P* = 0.0107] but not menthol concentration in both outcomes (Figures 4A,B). Interactions between all three factors (age, sex, and menthol concentration) were not significant for either nicotine intake or preference. However, an interaction of age and sex factors was reported for both outcomes [F_200μ*g/mL:intake:agexsex*_ (1, 86) = 4.609, *P* = 0.0346; F_200μ*g/mL:preference:agexsex*_ (1, 86) = 23.25, *P* < 0.0001] and interaction of concentration and age [F_200μ*g/mL:preference:concentrationxage*_ (4, 344) = 5.050, *P* = 0.0006] and concentration and sex F_200μ*g/mL:preference:concentrationxsex*_ (4, 344) = 2.464, *P* = 0.0449] factors were only reported for nicotine preference. A Tukey *post hoc* test revealed significant age-related effects in nicotine intake between adolescent and adult male animals only at 0 μg/mL (Figure 4A), and at all concentrations for menthol preference (Figure 4B). No significant age-related effects were observed for adolescent versus adult female animals in either outcome. On sex-related differences as reported by the RM three-way ANOVA, a Tukey *post hoc* test revealed only a significant difference in nicotine preference between male and female adolescent animals at 120 μg/mL (Figure 4D). No significant effects of sex were reported at any other concentrations for adolescent animals in nicotine intake (Figure 4C) or adult animals in nicotine intake or preference (Figures 4E,F).

### Systemic Administration of Menthol Does Not Enhance Oral Nicotine Consumption in Adult DBA/2J Mice

**FIGURE 5:**
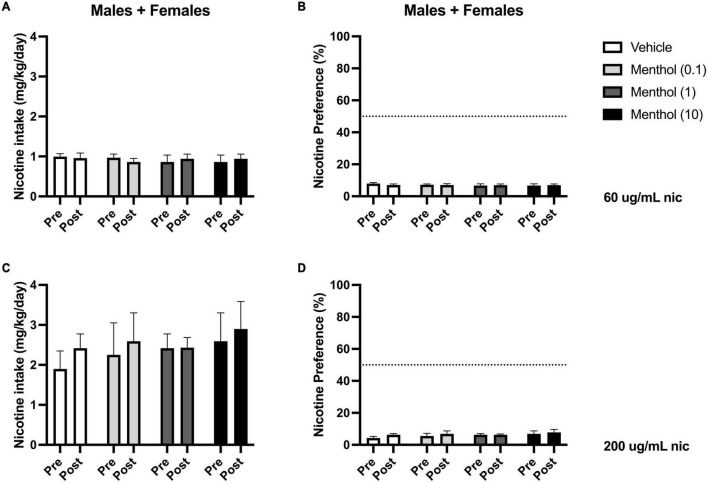
Impact of systemic administration of menthol on oral nicotine consumption in adult DBA/2J mice. Male and female D2J mice were given a choice of DI water or nicotine solution [60 or 200 μg/mL] for 7 days. On days 1–5, mice were poked daily to simulate i.p. injections. On day 6 (PRE), mice received an acute injection of vehicle (1:1:18) or menthol (0.1, 1, and 10 mg/kg). Nicotine intake and preference were then measured 24 h. after injection on day 7 (POST). **(A,C)** Nicotine intake and **(B,D)** preference 24 h. after drug administration was calculated. Data are presented as the mean ± SEM of 10–20 mice/group.

Next, we investigated the impact of systemic administration of menthol on oral nicotine consumption only in adult D2J mice. This was performed to determine if non-orosensory factors could contribute to menthol’s effects on nicotine consumption. D2J mice are known to have an aversion for nicotine, oral or otherwise; consequently, we investigated whether systemic administration of menthol would be sufficient to overcome their aversion to nicotine and enhance oral nicotine consumption. Animals were administered i.p. menthol while subjected to nicotine 2BC for six days, then an acute injection of menthol was administered. We report that systemic administration of menthol at all doses tested (0.1, 1, or 10 mg/kg, i.p.) failed in enhancing oral nicotine consumption in adult D2J mice (Figure 5). In addition, a RM two-way ANOVA revealed no significant effects of sex or menthol treatment on nicotine intake or preference at 60 or 200 μg/mL nicotine. Since no sex differences were observed, the data were pooled to form a mixed-sex cohort.

### Nicotine 2BC Concentration-Response in DBA/2NCrl Mice

To determine the potential role of the TAAR1 gene in menthols effects on oral nicotine consumption, we first investigated its effects on oral nicotine consumption in D2N mice, a DBA/2 substrain that possesses unmutated TAAR1. First, we determined a concentration-response of nicotine intake and preference in female and male adult D2N mice exposed to a range of nicotine concentrations (10–240 μg/mL). While nicotine intake increased with increasing nicotine concentration, preference for nicotine decreased. Furthermore, there were no observable sex differences in nicotine preference; however, females displayed a slightly greater intake than males at 60 and 120 μg/mL. A RM two-way ANOVA with sex and nicotine concentration as factors revealed significant effects of sex and concentration on nicotine intake [F_*intake:sex*_ (1, 58) = 16, *P* = 0.0002; F_*intake:concentration*_ (2.357, 136.7) = 89.45, *P* < 0.0001; Supplementary Figure 1A] but only an effect of concentration on nicotine preference [F_*preference:concentration*_ (1.726, 100.1) = 51.29, *P* < 0.0001; Supplementary Figure 1B]. No interaction of sex and concentration factors was reported for nicotine intake or preference outcomes. A Dunnett’s *post hoc* test revealed significant decreases in nicotine intake at 60, 120, and 240 μg/mL compared to the lowest concentration tested (10 μg/mL) for females, but only at 120 and 240 μg/mL for male animals. For menthol preference, the *post hoc* test also revealed significant decreases in nicotine preference at all concentrations tested for male and female animals compared to the 10 μg/mL concentration. A Sidak *post hoc* test showed significant sex differences at 60 and 120 μg/mL for sex-related effects in nicotine intake. No differences were observed in total fluid intake between male and female animals (Supplementary Figure 1C).

### Menthol Does Not Enhance Oral Nicotine Consumption in Adult DBA/2NCrl Mice

We then investigated menthol’s effects on oral nicotine consumption in D2N mice to assess the contributions of the TAAR1 mutation. Like D2J mice, menthol failed to enhance oral nicotine consumption in adult D2N mice. Similarly, it seemed to decrease rather than increase consumption. A RM two-way ANOVA with sex and menthol concentration as factors revealed significant effects of sex and concentration on nicotine intake [F_*intake:sex*_ (1, 58) = 6.873, *P* = 0.0112; F_*intake:concentration*_ (1.963, 113.8) = 15.62, *P* < 0.0001; Supplementary Figure 2A] and only an effect of concentration on nicotine preference [F_*preference:concentration*_ (1.822, 105.7) = 11.20, *P* < 0.0001; Supplementary Figure 2B]. No interaction of sex and concentration factors were reported for nicotine intake or preference. A Dunnett’s *post hoc* test revealed significant decreases in menthol intake at 10 and 60 μg/mL for female animals but only at 60 μg/mL for male animals. For menthol preference, the *post hoc* test revealed a significant decrease in menthol preference at 10 and 60 μg/mL for female animals but not in male animals at any of the concentrations tested. For sex-related differences, a Sidak *post hoc* test showed significant sex differences at 0 μg/mL but not at 10 or 60 μg/mL. Total fluid intake between male and female mice was similar and statistical differences were observed (Supplementary Figure 2C).

### Initial Preference for Nicotine Does Not Determine Menthol’s Effects on Oral Nicotine Consumption

BXD mice with differing initial preferences to oral nicotine (60 μg/mL) were used to examine if the initial basal preference for nicotine (low, moderate, or high) played a role in the effects of menthol on oral nicotine consumption. Consequently, six BXD lines (BXD53/2RwwJ, BXD55/RwwJ, BXD62/RwwJ, BXD64/RwwJ, BXD65/RwwJ, and BXD65a/RwwJ) (*n* = 3/genotype) were chosen and subjected to the 2BC paradigm. Individual preferences ranged from 10 to 90% [BXD53/2RwwJ (∼35%), BXD55/RwwJ (∼10%), BXD62/RwwJ (∼10%), BXD64/RwwJ (∼70%), BXD65/RwwJ (∼90%), and BXD65a/RwwJ (∼50%)] (Supplementary Figure 3A). In the six BXD lines tested, we found no significant correlation between basal nicotine preference and the effects of menthol. Statistical analysis according to a Friedman non-parametric test revealed no significant effects of menthol concentration on nicotine preference in most lines; however, it decreased nicotine preference significantly in the BXD65 line at 30 μg/mL and increased nicotine preference in the BXD65 line only at 90 μg/mL (Supplementary Figure 3B).

### Menthol Augments Nicotine CPP in C57BL/6J Mice but Not in DBA/2J Mice

**FIGURE 6:**
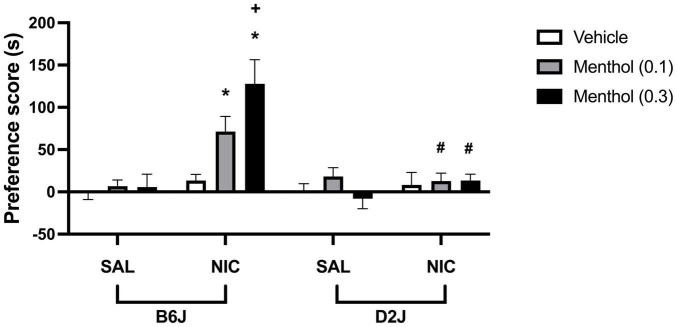
Effects of menthol on nicotine CPP in adult C57BL/6J and DBA/2J mice. Adult male and female mice were conditioned with either saline or nicotine (0.1 mg/kg, s.c.) for 3 days in the CPP paradigm. On each conditioning day, mice were pretreated with menthol (0.1 or 0.3 mg/kg, i.p.) or its vehicle (1:1:18) 30 min before nicotine or saline injection. Data are presented as the mean ± SEM of 10 mice/genotype/group. Results are based on an ordinary three-way ANOVA with strain, nicotine treatment, and menthol treatment as factors. *P < 0.05 vs. saline/veh, ^+^*P* < 0.05 vs. nicotine/veh and ^#^*P* < 0.05 vs. B6J. sal, saline; nic, nicotine.

To investigate the effects of menthol on nicotine conditioned reward in mice, adult B6J and D2J mice were conditioned with either saline or a low dose of nicotine (0.1 mg/kg, s.c.) for three days in the CPP paradigm. On each conditioning day, animals were pretreated with menthol (0.1 or 0.3 mg/kg, i.p.) or its vehicle (1:1:18) 30 min before nicotine or saline injection. Nicotine treatment did not induce significant CPP at the dose tested; however, menthol augmented nicotine CPP in B6J mice in a dose-dependent manner. This effect was strain dependent. Menthol treatment in D2J mice did not produce a parallel augmentation of nicotine CPP as was observed in B6J mice. According to an ordinary three-way ANOVA with strain, menthol treatment and nicotine treatment as factors, there were significant effects of all factors on nicotine CPP [F_*strain*_ (1, 108) = 14.33, *P* = 0.0003; F_*menthol*_ (2, 108) = 5.008, *P* = 0.0083, F_*nicotine*_ (1, 108) = 22.59, *P* < 0.0001], and an interaction of menthol treatment and strain factors [F_*mentholxstrain*_ (2, 108) = 5.357, *P* = 0.0060], menthol treatment and nicotine [F_*mentholxnicotine*_ (2, 108) = 5.263, *P* = 0.0066], strain and nicotine treatment [F_*strainxnicotine*_ (1, 108) = 14.14, *P* = 0.0003] and an interaction of all treatment [F_*strainxmentholxnicotine*_ (2, 108) = 3.093, *P* = 0.0494] (Figure 6). A Tukey *post hoc* analysis reveals robust CPP in nicotine-conditioned B6J mice pretreated with menthol at 0.1 and 0.3 mg/kg, but not in nicotine-conditioned mice treated with vehicle. No effects of nicotine, menthol, or its interactions were observed in D2J mice.

### Menthol Does Not Augment Nicotine-Induced Antinociception in the Hot Plate Test

**FIGURE 7:**
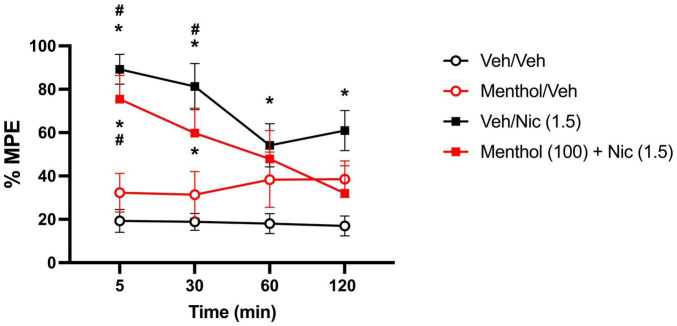
Effects of menthol on nicotine’s effects in the hot plate test in DBA/2J mice. Adult male and female D2J mice were pretreated for 30 min with either menthol (100 mg/kg, i.p.) or vehicle (1:1:18), then received nicotine (1.5, s.c.) or vehicle (saline) and were tested at 5-, 30-, 60- and 120-min post-injection. Antinociceptive response was calculated as percent maximum possible effect (% MPE). Data are expressed as the mean ± SEM of 10 mice/group. Results are based on a RM two-way ANOVA with time and treatment as factors. *P < 0.05 compared with veh/veh group. ^#^*P* < 0.05 compared with menthol/veh group. veh, vehicle; nic, nicotine.

Menthol was evaluated for its ability to enhance nicotine-induced antinociception in D2J mice in the hot-plate test. For that, we examined the impact of menthol (100 mg/kg, i.p.) on the time course of nicotine’s (1.5 mg/kg, s.c.) antinociceptive effects (Alsharari et al., 2015). According to a RM two-way ANOVA, significant effects of time [F_*time*_ (2.668, 96.07) = 3.131, *P* = 0.0343] and treatment [F_*treatment*_ (3, 36) = 16.29, *P* < 0.0001] were observed. Nicotine alone and nicotine + menthol induced significant and time-dependent antinociceptive effects in the tail-flick test compared to the vehicle-only group, with effects persisting up till 120 or 30 min, respectively (Figure 7). Menthol did not enhance nicotine-induced antinociception; rather, it showed a diminishing effect on nicotine-induced antinociception. In support, the AUC_0–120*min*_ for the nicotine + menthol group was significantly lower than the AUC_0–120*min*_ for the nicotine alone group (Table 1). Menthol alone also induced significant antinociceptive effects compared to the vehicle alone group. However, significantly higher AUC_0–120*min*_ were observed for the nicotine only and nicotine + menthol groups compared to the menthol control group.

**TABLE 1:** Effects of menthol pretreatment on nicotine-induced antinociception in the hot plate test.

Treatment	AUC_0–120*min*_ (mean ± SEM)
Veh/Veh	55.02 ± 17.37
Menthol/Veh	105.10 ± 42.00*
Veh/Nic	210.37 ± 37.00^*#^
Menthol/Nic	161.40 ± 47.17^*#+^

Data are expressed as the mean ± SEM of the AUC_0–120 min_ of the % MPE. N = 10 mice/group. Results are based on an ordinary one-way ANOVA. *P < 0.05 compared to veh/veh. ^#^P < 0.05 compared to menthol/veh. ^+^P < 0.05 compared to veh/nic. nic, nicotine; veh, vehicle.

### Effects of Acute MLA on Oral Nicotine Consumption in DBA/2J Mice

To examine the role of the α7 nAChR subunit in menthol’s effects on oral nicotine consumption, adult male and female D2J mice were given a choice of DI water or nicotine (10 μg/mL) for five days. On day 5, mice received a subcutaneous injection of saline or the selective α7 nAChR antagonist, methyllycaconitine (10 mg/kg) in the AM and PM 4 h apart. Nicotine consumption was then measured after 24 h (Supplementary Figure 4). No significant differences in oral nicotine consumption were observed in D2J mice for nicotine intake (*P* = 0.6839) or preference (*P* = 0.9305) after MLA administration compared to the saline-treated group.

## Discussion

Overall, our results revealed that the effects of menthol on oral nicotine consumption and reward are dependent on the genetic background of the animal, which supports the existence of genotype-specific mechanisms that may contribute to the variable effects of menthol in different populations. In adult and adolescent D2J mice, menthol failed to enhance oral nicotine consumption at low, moderate, and high nicotine concentrations (Figures 2–4). Rather than enhance oral nicotine consumption in D2J mice, menthol caused a further decrease at specific doses. Moreover, its effects were not due to age, sex, or an aversion to oral menthol (Figure 1). This is salient considering that we have previously shown that menthol enhances oral nicotine consumption in a sex-, concentration- and age-dependent manner in B6J mice (Bagdas et al., 2020). In addition, and in contrast to B6J mice, menthol failed to enhance nicotine conditioned reward and antinociception in D2J mice. To our knowledge, this is the first report to demonstrate genotypic differences in the effects of menthol.

Adolescent and adult female D2J animals, in general, had a greater preference for menthol (40–50%) compared to male animals (Figure 1). This is also paralleled in the clinic; women who smoke are more likely to use menthol flavored cigarettes than men who smoke (Smith et al., 2017). In the presence of nicotine at the low concentration (10 μg/mL), they maintained a similar preference level and menthol did not enhance nicotine consumption (Figure 2); however, with an increase in nicotine concentration, they showed a decrease both in their intake and preference (Figures 3, 4). The addition of menthol was insufficient in enhancing oral nicotine consumption or reverting their drinking profile to what it once was with menthol alone. Similarly, although male animals showed an aversion for menthol from the onset, with a preference range of 20–30% for adult and adolescent animals; in the presence of nicotine, preference levels fell even greater to less than 20%, except at the low nicotine concentration, where they maintained similar preference levels to that of menthol alone. This suggests that regardless of the nicotine concentration, menthol fails to enhance oral nicotine consumption or reduce nicotine aversion in D2J mice and that this lack in effect does not seem to be influenced by their sensitivity to menthol.

Many factors could explain the differences in menthol’s effects in B6J and D2J strains. A primary mechanism would be differences in menthol’s pharmacological targets between the two strains. In general, menthol has been demonstrated to facilitate nicotine intake in humans by blunting the oral aversive sensory experience to nicotine in cigarette smoke (Willis et al., 2011), an effect thought to be primarily mediated by the activation of the cold-sensitive receptors, transient receptor potential Melastatin 8 (TRPM8). This receptor has also been shown to play a role in oral aversion to nicotine in B6J mice (Fan et al., 2016). Indeed, TRPM8 is expressed by taste cells in the pharynx and tongue of Wistar rats (Abe et al., 2005; Sato et al., 2013). Menthol also has several other targets, including another member of the TRP family, the transient receptor potential Ankyrin 1 (TRPA1) receptor. The TRPA1 receptor has been shown to modulate the irritant effects of menthol at higher concentrations (Lemon et al., 2019). Menthol engages TRPA1 receptors in a bimodal fashion, activating the receptor at submicromolar to low micromolar concentrations and antagonizing it at higher concentrations (Karashima et al., 2007). While it is unknown if TRPM8 and TRPA1 receptor expression levels and function in oral and craniofacial structures differs between B6J and D2J mice, TRPM8 and TRPA1 mRNA expression do not differ between B6J or D2J animals in the striatum or the lungs (GeneNetwork.org- data not shown).

Menthol has also been demonstrated to modulate several nAChRs, including α4β2, α3β4, and α7 receptors where it acts as a negative allosteric modulator (NAM) (Hans et al., 2012; Ashoor et al., 2013). α7 nAChRs have been implicated in nicotine reinforcement and dependence (Nomikos et al., 2000; Besson et al., 2012) such that agonism of α7 nAChRs results in an aversive phenotype for nicotine while antagonism results in a nicotine-seeking phenotype (Brunzell and McIntosh, 2012; Harenza et al., 2014). We have previously shown that α7 nAChRs play a modulatory role in the effects of menthol on oral nicotine consumption (Bagdas et al., 2020). α7 KO mice on a B6J background show an increased intake and preference for nicotine alone compared to their WT counterparts; however, mice null for the same subunit demonstrate a decreased intake and preference for nicotine with menthol present compared to nicotine alone. Perhaps the most exciting discovery is that B6J and D2J differ in basal α7 mRNA expression in the NAc and that basal α7 mRNA expression in the NAc is inversely correlated with nicotine place conditioning in BXD mice (Harenza et al., 2014). Indeed, this increase in basal α7 mRNA tone in the NAc of D2J mice may contribute to their nicotine avoidant phenotype. However, when we evaluated the effects of acute MLA administration (10 mg/kg) on oral nicotine consumption, it was insufficient in reverting the nicotine avoidant phenotype in D2J mice (Supplementary Figure 4). This dose has been shown to significantly revert nicotine CPP in mice that were treated with PHA-543613, an α7 nAChR-selective agonist (Harenza et al., 2014).

One might argue then that the differential effect of menthol in B6J and D2J mice could be due to D2J’s basal aversion to oral nicotine, such that D2J mice are very resistant to nicotine and that menthol is unable to overcome this aversion. To investigate this possibility, we used two approaches. In the first approach, we bypassed the orosensory route by administering menthol systemically and then examined its effects on oral nicotine consumption in D2J mice. We have shown previously that an acute i.p. administration of menthol was sufficient in enhancing oral nicotine consumption in B6J mice (Bagdas et al., 2020). This indicated that both orosensory-dependent and independent mechanisms were at play for B6J mice. Here, we found that systemic administration of menthol (0.1, 1, or 10 mg/kg) failed to enhance oral nicotine consumption in adult male or female D2J mice (Figure 5), strengthening the idea that genetic background plays a significant role in sensitivity to the effects of menthol on nicotine intake and thus, demonstrates that the lack of effect of menthol in D2J mice is not due to orosensory aversion alone. In the second approach, we investigated if the effects of menthol depend on basal preference for nicotine. Consequently, we examined the impact of menthol on oral nicotine consumption in mice with differing basal preferences for nicotine in the 2BC paradigm. Adult female BXD mice from different lines (BXD53/2RwwJ, BXD55/RwwJ, BXD62/RwwJ, BXD64/RwwJ, BXD65/RwwJ, and BXD65a/RwwJ) with either a low, moderate, or high basal preference for nicotine (60 μg/mL) were subjected to nicotine 2BC with menthol (30 and 90 μg/mL). We found no apparent correlations between initial nicotine preference and the effects of menthol. In most lines (BXD53, 55, 62, and 64), menthol had no effect on nicotine preference; however, it decreased nicotine preference significantly in the BXD65 line and increased nicotine preference in the BXD65a line at specific concentrations (Supplementary Figure 3).

Our investigative efforts into uncovering the underlying mechanisms of menthol’s differential effects on nicotine between the B6J and D2J strains led us to assess menthol’s effects in a model of acute reward. Systemic administration of menthol has been demonstrated to augment nicotine CPP and nicotine reward-related behaviors in mice (Henderson et al., 2017). However, it is unknown if systemic administration of menthol would also augment nicotine CPP in D2J mice. While genetic background influences nicotine-induced CPP and aversion in mice (Ise et al., 2014; Kutlu et al., 2015), to our knowledge, no one has assessed if genotypic factors extend to menthol’s effects on nicotine CPP. We, therefore, examined menthol’s effects on acute reward to nicotine in the CPP test in B6J and D2J mice. We found that menthol augmented nicotine CPP differentially in B6J mice compared to D2J mice (Figure 6). Menthol dose-dependently enhanced nicotine CPP in B6J mice but had no effect in D2J mice. Nicotine kinetics between both strains does not seem to play a significant role in the differential effects of menthol. Both strains demonstrate similar elimination half-lives for nicotine, except in cotinine metabolism, where D2J mice show significantly slower clearance (Siu and Tyndale, 2007). However, cotinine is not known to be pharmacologically active after systemic administration and fails to induce CPP in rats (Fudala and Iwamoto, 1986). We then investigated if these differences extended to other pharmacological actions of nicotine, like its antinociceptive effects in the hot plate test. We have previously reported that menthol enhances nicotine-induced antinociception in B6J mice (Alsharari et al., 2015). However, to our knowledge, menthol’s effects on nicotine-induced antinociception have not been assessed in D2J mice. Here, we find that menthol diminished rather than prolonged the antinociceptive effects of nicotine in D2J mice (Figure 7). Menthol decreased the AUC_0–120 *min*_ of nicotine’s antinociceptive effects relative to the nicotine-only treated group (Table 1). A surprising finding considering that a prominent effect of menthol in relation to nicotine is its ability to decrease nicotine metabolism (Alsharari et al., 2015; Ha et al., 2015). Now while nicotine metabolism has been compared between the B6J and D2J strains, a key limitation in our understanding involves the metabolism of menthol and if that differs between both strains. This could provide more insight into the differential effects of menthol in these strains.

Lastly, we assessed the role of the TAAR1 gene in nicotine intake. TAAR1 has been found to play a significant role in regulating dopamine, norepinephrine, and serotonin neurotransmission in the CNS (Rutigliano et al., 2018). D2J mice possess a non-synonymous mutation in this gene, resulting in a non-functional allele that imparts a phenotype for increased methamphetamine (MA) drinking compared to other DBA substrains. DBA mice from vendors like Charles River (DBA/2NCrl), Envigo (DBA/2NHsd), or Taconic (DBA/2NTac) have not been shown to possess such a mutation or consequent MA phenotype (Reed et al., 2018). However, it is unknown if this mutation plays a role in aversion to oral nicotine. Consequently, we subjected DBA/2NCrl mice to the nicotine 2BC paradigm. Likewise, menthol failed to enhance oral nicotine consumption in these mice at a low concentration of nicotine (10 μg/mL) (Supplementary Figure 2). Our results, therefore, suggest that the spontaneous mutation in the TAAR1 gene that has been found to impact methamphetamine drinking in D2J mice does not play a role in menthol’s effects on oral nicotine consumption in mice.

## Conclusion

We show that genetic background plays a significant role in sensitivity to menthols’ effects on oral nicotine consumption, acute reward, and nicotine-induced antinociception. To our knowledge, we are the first to show that menthol fails to enhance oral nicotine consumption, augment nicotine CPP or enhance nicotine-induced antinociception in D2J mice, thus demonstrating genotypic differences in the effects of menthol on nicotine. Furthermore, the lack of effect on nicotine consumption in D2J mice was not due to nicotine concentration, oral aversion to menthol, basal preference to nicotine, age, sex, or the TAAR1 receptor. Menthol’s differential effects on antinociception and CPP are likely not due to cotinine metabolism but may be due to differential alterations in menthol metabolism between both strains.

## Data Availability Statement

The original contributions presented in this study are included in the article/Supplementary Material, further inquiries can be directed to the corresponding author.

## Ethics Statement

The animal study was reviewed and approved by Institutional Animal Care and Use Committee of Virginia Commonwealth University.

## Author Contributions

MD and LA: conceptualization, methodology, and project administration. LA, YR, OO, and JG: formal analysis. LA, YR, OO, JG, AJ, and DB: investigation. LA, YR, and OO: writing—original draft. LA, MD, AJ, and DB: writing—review and editing. MD: funding acquisition. All authors contributed to the article and approved the submitted version.

## Author Disclaimer

This content is solely the authors’ responsibility and does not necessarily represent the official views of the NIH or the FDA.

## Conflict of Interest

The authors declare that the research was conducted in the absence of any commercial or financial relationships that could be construed as a potential conflict of interest.

## Publisher’s Note

All claims expressed in this article are solely those of the authors and do not necessarily represent those of their affiliated organizations, or those of the publisher, the editors and the reviewers. Any product that may be evaluated in this article, or claim that may be made by its manufacturer, is not guaranteed or endorsed by the publisher.
