# Supplementary material for: Genotypic Differences in the Effects of Menthol on Nicotine Intake and Preference in Mice
Source: Front Neurosci. 2022 Jun 13;16:905330. doi: 10.3389/fnins.2022.905330 (PMC9234577; doi:10.3389/fnins.2022.905330)

Supplementary Material

Supplementary Figure 1. Oral nicotine consumption in adult male and female DBA/2NCrl mice. Adult male and female DBA/2NCrl mice were given a choice of DI water or nicotine (10-240 µg/mL) in the two-bottle choice paradigm. The nicotine concentration was increased every three days, and the averaged values from all three days were used to calculate nicotine intake and preference. **(A)** Average nicotine intake (mg/kg/day), **(B)** nicotine preference (%), and **(C)** total fluid intake (mL/day) are shown. Data are presented as the mean ± SEM of 10 mice/sex. Results are based on a RM two-way ANOVA with sex and menthol concentration as factors. *p < 0.05 vs 10 µg/mL solution. #p < 0.05 vs males.

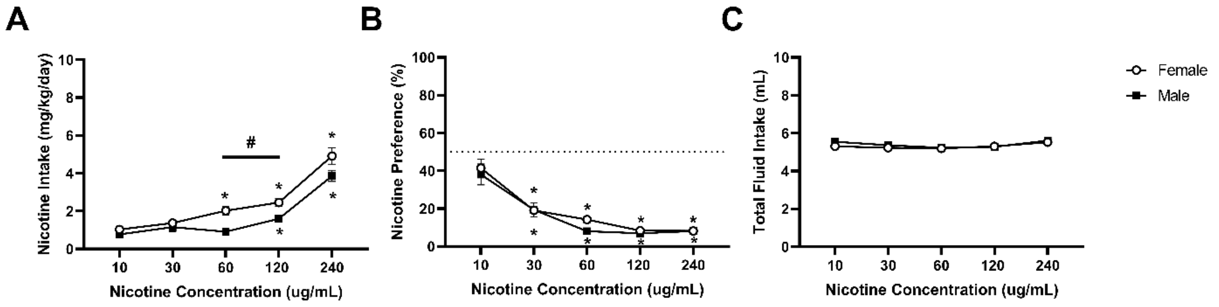

Supplementary Figure 2. Effects of menthol on oral nicotine consumption in adult DBA/2NCrl mice. Adult male and female mice were given a choice of DI water or menthol + nicotine. Nicotine was kept constant at 10 µg/mL while menthol concentration was varied (0, 10 & 60 µg/mL). **(A)** Averaged nicotine intake (mg/kg/day), **(B)** nicotine preference (%), and **(C)** total fluid intake (mL/day) are presented. Data are expressed as the mean ± SEM of 10-15 mice/sex. Results are based on a RM two-way ANOVA with sex and menthol concentration as factors. *p < 0.05 vs 0 µg/mL. #p < 0.05 vs males.

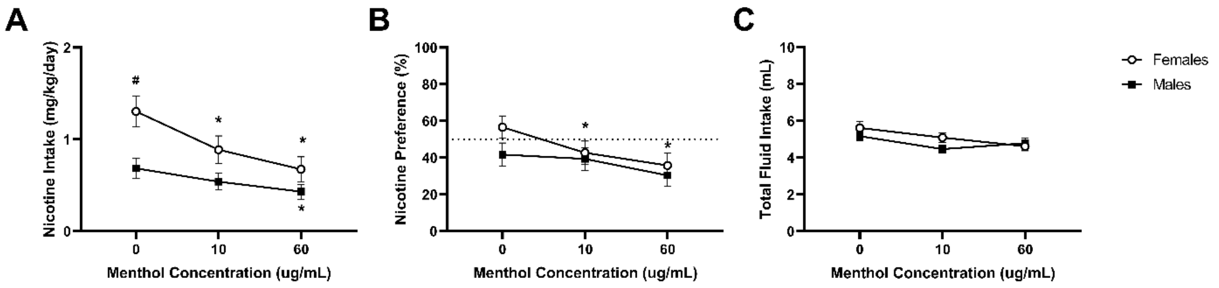

Supplementary Figure 3. Effects of oral menthol on oral nicotine consumption in BXD mice. Six adult female BXD mice from different lines with differing baseline preferences for nicotine were given a choice of DI water or menthol + nicotine. Nicotine was kept constant at 60 µg/mL while menthol concentration varied (0, 30 & 90 µg/mL) and increased every three days. **(A)** Initial preference for 60 µg/mL nicotine and **(B)** effects of menthol on nicotine preference. Data are expressed as the mean ± SEM of 3 mice/genotype. Results are based on a nonparametric Friedman test. *p < 0.05 vs 0 µg/mL within each strain.

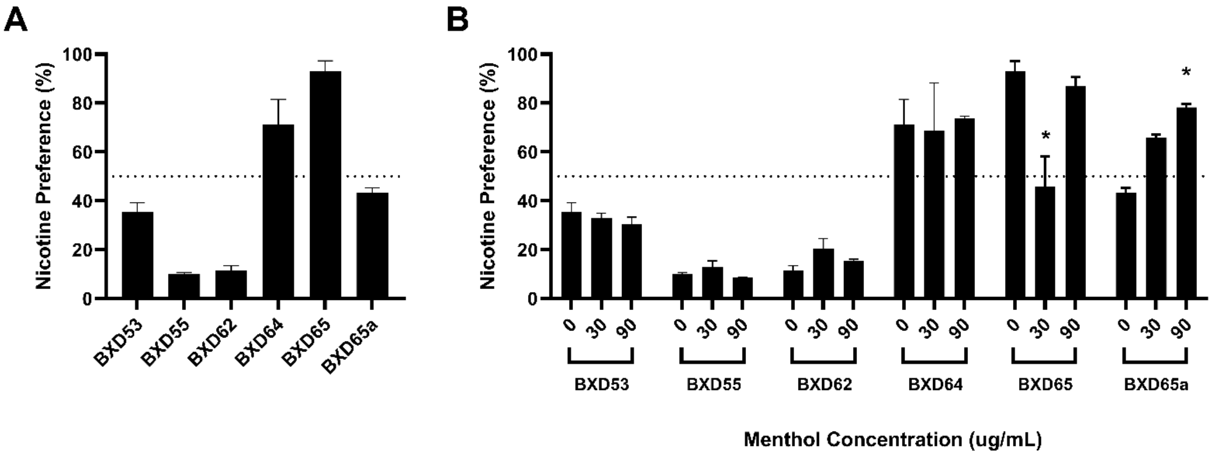

Supplementary Figure 4. Effect of acute MLA, a selective α7 nAChR antagonist, on oral nicotine consumption in DBA/2J mice. Adult male and female D2J mice were given a choice of DI water or nicotine (10 µg/mL) for five days. On day 5, mice received subcutaneous injections of either saline or MLA (10 mg/kg) in the AM and PM 4 hours apart. **(A)** Nicotine intake and **(B)** preference were then measured after 24 hours. Data are expressed as the mean ± SEM of 18-40 mice/group.

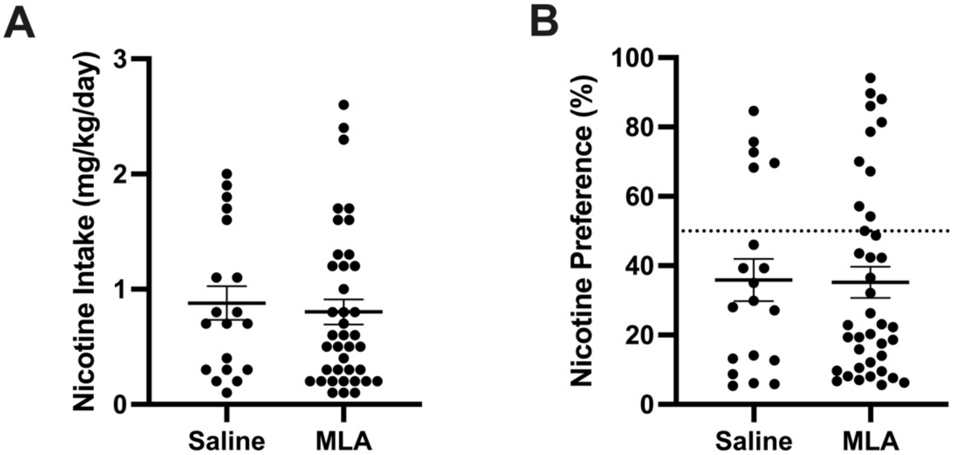

Supplement: Supplementary file 1 [file Data_Sheet_1.PDF]
